# Association Between Arterial Stiffness and Bone Microarchitectural Deterioration in Type 2 Diabetes: A Cross‐Sectional Study

**DOI:** 10.1111/1753-0407.70181

**Published:** 2025-12-10

**Authors:** Yangting Wang, Xiaoting Chen, Jinjian Xu, Qifeng Ying, Lizhi Liang, Xiaohong Wu

**Affiliations:** ^1^ Hangzhou Normal University Hangzhou Zhejiang China; ^2^ Geriatric Medicine Center, Key Laboratory of Endocrine Gland Diseases of Zhejiang Province, Department of Endocrinology Zhejiang Provincial People's Hospital (Affiliated People's Hospital, Hangzhou Medical College) Hangzhou Zhejiang China; ^3^ Department of Osteoporosis Diagnosis and Treatment Center Zhejiang Provincial People's Hospital (Affiliated People's Hospital, Hangzhou Medical College) Hangzhou Zhejiang China

**Keywords:** arterial stiffness, bone microarchitectural degeneration, bone mineral density, bone trabecular score, pulse wave conduction velocity, type 2 diabetes mellitus

## Abstract

**Background:**

Trabecular bone score (TBS) and brachial‐ankle pulse wave velocity (baPWV) are established indicators of bone microstructure and arterial stiffness. However, their association has not been explored in the context of type 2 diabetes (T2DM). We aimed to investigate the factors influencing bone microarchitectural deterioration in patients with T2DM, examining the associations of baPWV with both TBS and bone mineral density (BMD).

**Materials and Methods:**

This study comprised 646 participants with T2DM. L1–L4 TBS was assessed via dual‐energy X‐ray bone densitometry and categorized into three groups. Independent risk factors influencing L1–L4 TBS in T2DM patients were examined by stepwise linear regression. The independent association between baPWV and TBS was further analyzed using linear regression with sequential adjustment, while the relationship between baPWV and BMD was assessed using univariable and multivariable linear regression.

**Results:**

Age, Body Mass Index (BMI), Urinary Albumin‐to‐Creatinine Ratio, diastolic blood pressure, Total Type I collagen N‐terminal Propeptide, Alkaline Phosphatase, and baPWV were identified as independent risk factors for L1–L4 TBS. In a series of progressively adjusted models, baPWV remained significantly and independently negatively associated with L1–L4 TBS (*β* = −0.076, 95% CI: −0.114, −0.038). Furthermore, when baPWV exceeded 1531 cm/s, the risk of degraded bone microarchitecture significantly increased. However, baPWV showed no significant independent association with BMD after multivariable adjustment (*p* > 0.05).

**Conclusion:**

In patients with T2DM, baPWV demonstrated an independent inverse association with L1–L4 TBS, suggesting a potential link between arterial stiffness and bone microarchitectural deterioration and offering a new clinical perspective on the bone–vascular axis.

## Highlights

- Identified baPWV as a novel independent influencing factor of bone microarchitecture deterioration in T2DM, elucidating a bone–vascular axis.
- Beyond vascular factors, the study identifies age, BMI, UACR, DBP, TPINP, and ALP as independent determinants of L1–L4 TBS.
- Proposes baPWV as a practical indicator for simultaneous cardiovascular and fracture risk assessment in diabetic patients.

## Graphical abstract

Identified baPWV as a novel independent influencing factor of bone microarchitecture deterioration in T2DM, elucidating a bone–vascular axis. Proposes baPWV as a practical indicator for simultaneous cardiovascular and fracture risk assessment in diabetic patients.

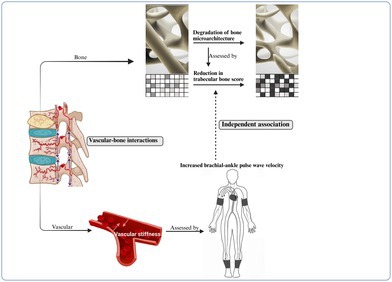

## 1 Introduction

Diabetes mellitus (DM) and osteoporosis, prevalent metabolic disorders, interact and influence one another [1]. The presence of osteoporosis dramatically increases the incidence of disability and mortality in patients with diabetes, which seriously affects their quality of life [2]. Osteoporosis is a condition defined by diminished bone mass and microstructural deterioration of bone tissue, with diagnosis relying on bone mineral density (BMD) assessed by dual‐energy X‐ray absorptiometry (DXA) [3]. However, the discrepancy between increased bone mass and fracture incidence suggests that the changes in BMD do not accurately reflect alterations in bone metabolism in patients with type 2 diabetes (T2DM) [4]. Consequently, a new index is required to more accurately reflect the bone issues in patients with T2DM and to guide the evaluation of fracture risk.

The trabecular bone score (TBS), a gray‐scale texture measurement based on DXA, is an important indicator of bone microarchitecture, which can be used to assess the quality and strength of bones [5]. A few studies concluded that TBS predicts osteoporotic fractures, at least partially independent of BMD and clinical risk factors [6]. Previous research reported a substantial reduction in TBS in patients with T2DM [7], whereas BMD, conversely, rises [4]. This may relate to patients with T2DM, who typically exhibit hypo‐osteoconverting osteoporosis, characterized by decreased osteoblast activity and impaired bone repair, making it difficult to detect through BMD [4]. However, the majority of research has been on BMD, with less emphasis on the determinants affecting TBS.

On the other hand, increased arterial stiffness represents an important pathological mechanism contributing to elevated cardiovascular risk and adverse outcomes in diabetic patients [8]. Brachial‐ankle pulse wave velocity (baPWV) serves as a reliable and practical measure of arterial stiffness and is widely used in both clinical and research settings [8]. In recent years, the conceptual framework of the “bone–vascular axis” has gained increasing attention, providing new insights into the shared pathological mechanisms linking bone and vascular systems in diabetes [9]. Although previous studies have reported an association between baPWV and BMD in general populations [10], this relationship has not been validated in patients with T2DM. More importantly, the association between baPWV and TBS, a specific indicator of bone microarchitecture, remains unexplored.

Therefore, this study aims to systematically analyze factors influencing the TBS in patients with T2DM, with a specific focus on examining the associations of baPWV with TBS and BMD. Clarifying these relationships could offer new insights for the early identification of patients with T2DM at elevated risk of bone microstructural deterioration.

## 2 Methods

### 2.1 Study Participants

This research was a cross‐sectional analysis. The study cohort included 646 patients with T2DM treated in the Department of Endocrinology at Zhejiang Provincial People's Hospital from November 2022 to March 2025. Criteria for inclusion: The diagnosis of DM was established according to the 2024 American Diabetes Association criteria: fasting blood glucose ≥ 7.0 mmol/L, 2‐h postprandial blood glucose ≥ 11.1 mmol/L, or glycated Hb ≥ 6.5%. Exclusion criteria: (1) individuals with incomplete data on bone trabecular score and arm and ankle pulse wave velocity during hospitalization; (2) patients with concurrent hyperthyroidism, malignant tumors, hyperparathyroidism, chronic kidney disease stage 4 or higher (eGFR < 30 mL/min/1.73 m^2^) or those who have received bisphosphonates, thyroxine, or glucocorticoids in the past year; (3) other types of DM.

### 2.2 Collection of Clinical Data and Laboratory Tests

Data on patients' names, genders, ages, medical histories, surgical histories, and medication histories were gathered, while physical examinations encompassed blood pressure, height, weight, waist circumference (WC), and body mass index (BMI) calculations. The participants removed their outer garments and footwear before height and weight measurements. Smoking and coffee intake were forbidden for 30 min prior to blood pressure assessment, and blood pressure in the left upper extremity was recorded following a minimum rest period of 5–10 min in a tranquil setting. Blood pressure was recorded every 5 min, and the mean value was determined as the final blood pressure reading of the study participants after three assessments.

Following an 8‐h fast, venous blood was obtained in the early morning of the subsequent day. High‐performance liquid chromatography was employed to quantify glycated hemoglobin (HbA1c). The rate method was utilized for the assessment of alanine aminotransferase (ALT), aspartate aminotransferase (AST), and alkaline phosphatase (ALP). The enzyme coupling rate method facilitated the measurement of blood urea nitrogen (BUN). The enzyme method was applied to determine serum creatinine (Scr), uric acid (SUA), triglycerides (TG), total cholesterol (TC), high‐density lipoprotein cholesterol (HDL‐C), and low‐density lipoprotein cholesterol (LDL‐C). The colorimetric method was implemented for Hb detection, while the Roche electrochemiluminescence method was used to measure parathyroid hormone (PTH). N‐terminal osteocalcin (N‐t OC), total type I collagen N‐terminal propeptide (TPINP), type I collagen carboxy‐terminal peptide beta (β‐CTX), 25‐hydroxyvitamin D (25(OH) D), and fasting plasma glucose (FPG) were quantified using the hexokinase method, whereas fasting insulin (FINS) and fasting C‐peptide (FCP) were assessed via chemiluminescence. The homeostasis model assessment of insulin resistance (HOMA‐IR) was calculated as follows: HOMA‐IR = fasting blood glucose (mmol/L) × fasting insulin (μ IU/mL)/22.5. The homeostasis model assessment of β‐cell function (HOMA‐β) was calculated as: HOMA‐β = 20 × fasting insulin (μ IU/mL)/(fasting blood glucose (mmol/L) − 3.5).

### 2.3 Measurement and Definitions of TBS and baPWV

A GE Lunar dual‐energy X‐ray bone densitometer was utilized to assess and document the femoral neck bone mineral density (FN BMD), total hip bone mineral density (TH BMD), and lumbar spine 1–4 BMD (L1–L4 BMD) of the patients. Additionally, TBS of lumbar spine DXA images was evaluated using TBS iNsight software (version 2.1.0.2). The ankle‐brachial index (ABI) and baPWV were concurrently assessed using an Omron BP‐203PREIII atherosclerosis detection apparatus. Rest for over 5 min prior to measurement, then assume a supine position with hands facing upward on either side of the body throughout the assessment. Measurements were conducted twice, with the second instance yielding the definitive value. This study utilized the average values of baPWV and ABI from both the left and right sides for analysis. According to previous studies, TBS ≤ 1.230 was considered degraded microarchitecture, 1.230 < TBS < 1.310 was partially degraded bone microarchitecture, and TBS ≥ 1.310 was normal [11]. baPWV < 1400 cm/s is classified as normal arterial stiffness, 1400 cm/s ≤ baPWV ≤ 1800 cm/s indicates elevated arterial stiffness, and baPWV > 1800 cm/s signifies severe arterial stiffness [12]. A *T* score ≥ −1.0 is considered normal bone density, −2.5 < *T* score < −1.0 indicates osteopenia, and *T* score ≤ −2.5 is diagnosed as osteoporosis. Additionally, according to the ISCD definition, a *Z* score ≤ −2.0 falls within the “below age‐specific expected range” category [13] and was classified as the osteopenia group in this study.

### 2.4 Statistical Analysis

All data were statistically analyzed utilizing SPSS 25.0 software, while GraphPad Prism 9.0 was employed for graphical representation. Measurements that follow a normal distribution were presented as mean ± standard deviation, and one‐way ANOVA was employed for group comparisons; non‐normally distributed measurements were represented as M (Q1, Q3), while the Kruskal–Wallis H‐test was utilized for group comparisons. Counts were presented as cases (%), and the chi‐square test was applied for group comparisons. Spearman's test was employed to examine the correlation between baPWV and L1–L4 TBS/BMD. Proportions of low TBS and osteoporosis were visualized using stacked bar charts by arterial stiffness group, with chi‐square tests evaluating group differences. baPWV and TBS were skewed‐distributed data. Following logarithmic transformation of baPWV and TBS and standardization of independent variables, stepwise linear regression analysis was employed to identify the independent correlates of L1–L4 TBS in individuals with T2DM. Subsequent multifactorial linear regression analyses were conducted across multiple models to investigate the relationship between baPWV and L1–L4 TBS. Perform univariable linear regression followed by multivariable linear regression to analyze the relationships between baPWV and BMD at the femoral neck, total hip, and lumbar spine. The Restricted Cubic Spline (RCS) plots for baPWV and L1–L4 TBS were illustrated. The predictive value of baPWV for the low TBS was assessed using the Receiver Operating Characteristics (ROC) curves analysis. The difference was statistically significant if *p* < 0.05.

## 3 Results

### 3.1 Baseline Characteristics

Out of 646 individuals selected, 231 patients exhibited a decrease in TBS. We divided the 646 patients into three groups based on their TBS values, and the characteristics were shown in Table 1. In comparison to the other two groups, the TBS ≤ 1.23 group exhibited significantly elevated levels of age, duration of diabetes, BUN, UACR, ALP, N‐t OC, TPINP, and baPWV, alongside comparatively diminished levels of DBP, BMI, Hb, eGFR, LDL‐C, FN BMD, TH BMD, L1–L4 BMD, and the male proportion. There was no difference in SBP, WC, HbA1c, HOMA‐IR, HOMA‐β, UA, Scr, TG, TC, HDL‐C, AST, ALT, Ca, P, PTH, β‐CTX, 25(OH)D, and ABI among the three groups.

**TABLE 1:** Demographic and clinical characteristics of T2DM patients stratified by TBS levels.

Characteristics	TBS ≤ 1.23 (*n* = 92)	1.23 < TBS < 1.31 (*n* = 139)	TBS ≥ 1.31 (*n* = 415)	*p*
Age (year)	62.50 (56.00,69.00)	59.00 (45.50,65.00)	49.00 (39.00,58.00)	< 0.001
Male (%)	32.61% (*n* = 30)	60.43% (*n* = 84)	79.52% (*n* = 330)	< 0.001
SBP (mmHg)	135.88 ± 20.74	133.92 ± 16.23	132.82 ± 17.34	0.306
DBP (mmHg)	75.50 (69.00,83.25)	78.00 (71.00,85.50)	80.00 (74.00,87.50)	0.002
BMI (kg/m^2^)	24.02 (21.39,26.57)	25.66 (22.88,27.99)	25.23 (23.40,27.63)	0.004
WC (cm)	93.00 (85.00,100.00)	95.00 (88.00,103.00)	93.00 (87.00,100.00)	0.098
Diabetic duration (month)	120.00 (12.75,192.25)	49.00 (1.00,129.00)	46.00 (1.00,120.00)	0.002
HbA1c (%)	9.25 (7.45,10.88)	9.10 (8.00,10.70)	9.65 (7.90,11.28)	0.194
HOMA‐IR	2.39 (1.40,4.06)	2.16 (1.36,3.72)	1.99 (1.14,3.51)	0.184
HOMA‐β	47.81 (27.73,112.55)	41.67 (20.32,92.51)	44.92 (25.20,88.63)	0.261
Hb (g/L)	134.00 (124.00,144.25)	138.50 (126.00,153.00)	146.00 (135.75,156.00)	< 0.001
eGFR (mL/min/1.73 m^2^)	93.48 (82.10,102.70)	100.53 (88.53,113.97)	106.74 (96.25,116.28)	< 0.001
Scr (μmol/L)	64.10 (50.82,77.80)	62.50 (53.70,74.80)	66.90 (56.25,79.17)	0.193
BUN (mmol/L)	5.83 (4.92,7.12)	5.34 (4.38,6.72)	5.13 (4.29,6.21)	< 0.001
UACR (mg/g.cr)	22.17 (11.65,50.39)	16.82 (8.83,41.38)	12.95 (7.44,31.41)	< 0.001
UA (μmol/L)	306.50 (245.25,376.75)	333.00 (277.50,387.50)	330.50 (272.25,400.75)	0.057
TG (mmol/L)	1.42 (1.01,2.07)	1.35 (1.02,2.19)	1.53 (1.03,2.13)	0.680
TC (mmol/L)	4.45 (3.60,5.19)	4.25 (3.64,5.13)	4.62 (3.75,5.23)	0.277
HDL‐C (mmol/L)	0.99 (0.87,1.16)	1.00 (0.84,1.12)	0.94 (0.82,1.08)	0.064
LDL‐C (mmol/L)	2.59 ± 0.77	2.59 ± 0.85	2.79 ± 0.85	0.015
ALT (U/L)	21.00 (13.00,36.75)	24.00 (16.00,33.00)	24.00 (17.00,40.75)	0.288
AST (U/L)	21.00 (19.00,29.00)	22.00 (17.25,28.00)	22.00 (18.00,29.00)	0.721
ALP (U/L)	80.00 (61.00,98.00)	81.00 (63.00,97.00)	74.00 (60.00,92.00)	0.048
Ca (mmol/L)	2.26 (2.20,2.33)	2.27 (2.20,2.35)	2.28 (2.22,2.34)	0.363
P (mmol/L)	1.23 (1.12,1.38)	1.23 (1.11,1.35)	1.21 (1.10,1.36)	0.746
PTH (pg/mL)	40.50 (29.70,53.95)	41.25 (33.00,49.23)	39.50 (31.30,52.10)	0.763
N‐t OC (ng/mL)	13.70 (9.95,18.35)	12.20 (9.67,15.48)	11.40 (9.10,14.50)	< 0.001
TPINP (ng/mL)	42.70 (33.60,61.50)	39.65 (30.47,51.35)	38.30 (28.40,47.90)	0.003
β‐CTX (pg/mL)	483.00 (315.70,700.40)	413.50 (275.50,539.00)	401.80 (272.30,593.00)	0.051
25 (OH)D (ng/mL)	17.49 (13.96,21.39)	17.70 (12.41,21.99)	15.72 (12.33,20.09)	0.063
ABI	1.10 (1.00,1.17)	1.11 (1.02,1.18)	1.08 (1.01,1.16)	0.210
baPWV (cm/s)	1656.25 (1450.62,1935.62)	1542.50 (1314.00,1791.25)	1433.00 (1284.25,1583.25)	< 0.001
FN BMD (g/cm^2^)				
*Z* score (*n* = 101)	−0.35 (−0.93,0.23) (*n* = 4)	−1.10 (−1.20,−0.20) (*n* = 17)	−0.10 (−0.83,0.32) (*n* = 80)	0.176
*T* score (*n* = 545)	−2.00 (−2.52,‐1.48) (*n* = 88)	−1.40 (−2.08,−0.90) (*n* = 122)	−0.80 (−1.40,−0.10) (*n* = 335)	< 0.001
TH BMD (g/cm^2^)				
*Z* score	−0.25 (−0.88,0.50)	−0.80 (−1.10,0.40)	−0.10 (−0.60,0.60)	0.171
*T* score	−1.30 (−2.10,−0.78)	−0.80 (−1.50,−0.32)	−0.20 (−0.80,0.60)	< 0.001
L1–L4 BMD (g/cm^2^)				
*Z* score	0.00 (−0.20,0.43)	−1.20 (−1.50,−0.70)	0.00 (−0.90,0.93)	< 0.001
*T* score	−2.20 (−2.80,−1.28)	−1.20 (−1.90,−0.28)	−0.30 (−1.15,0.65)	< 0.001

*Note:* Data are presented as median (interquartile range), mean ± standard deviation, or percentage (number) as appropriate. *p* values are derived from Chi‐square test, ANOVA or Kruskal–Wallis test based on data distribution.

Abbreviations: 25(OH)D: 25‐hydroxyvitamin D; ABI: Ankle‐Brachial Index; ALP: alkaline phosphatase; ALT: alanine aminotransferase; AST: aspartate aminotransferase; baPWV: brachial‐ankle pulse wave velocity; BMI: body mass index; BUN: blood urea nitrogen; Ca: calcium; DBP: diastolic blood pressure; eGFR: estimated glomerular filtration rate; FN BMD: femoral neck bone mineral density; Hb: hemoglobin; HbA1c: glycated hemoglobin; HDL‐c: high‐density lipoprotein cholesterol; HOMA‐IR: homeostatic model assessment insulin resistance; HOMA‐β: homeostatic model assessment of β‐cell function; L1–L4 BMD: lumbar spine 1 to 4 bone mineral density; LDL‐c: low‐density lipoprotein cholesterol; N‐t OC: N‐terminal osteocalcin; P: phosphorus; PTH: parathyroid hormone; SBP: systolic blood pressure; Scr: serum creatinine; TBS: trabecular bone score; TC: total cholesterol; TG: triglyceride; TH BMD: total hip bone mineral density; TPINP: total type I collagen N‐terminal propeptide; UA: uric acid; UACR: urinary albumin‐to‐creatinine ratio; WC: waist circumference; β‐CTX: type I collagen carboxy‐terminal peptide β.

### 3.2 baPWV Showed Correlations With Both TBS and BMD

**FIGURE 1:**
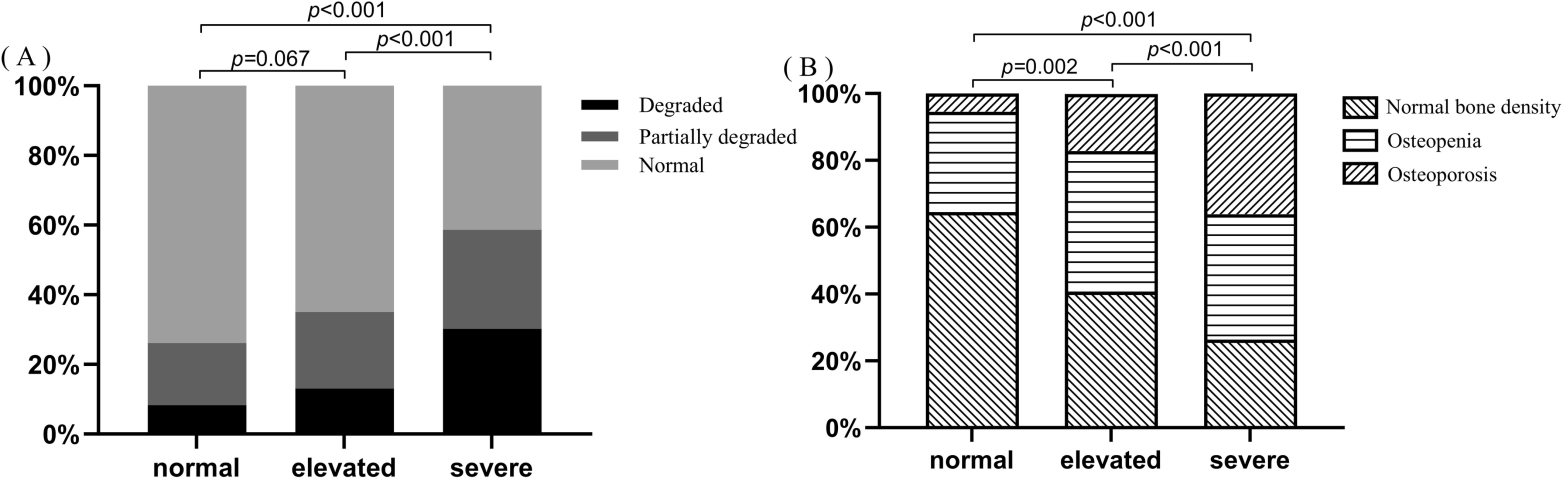
Proportional distribution of (A) TBS levels and (B) BMD categories by arterial stiffness severity. (A) Distribution of TBS categories (normal: TBS ≥ 1.31; partially degraded: 1.23 < TBS < 1.31; degraded: TBS ≤ 1.23) among T2DM patients with different arterial stiffness levels (normal: baPWV < 1400; elevated: 1400–1800; severe: > 1800), analyzed by chi‐square test. *p* values are labeled. (B) Prevalence of BMD categories (normal bone density: *T* score ≥ −1.0 or *Z* score > −2.0, osteopenia: −2.5 < *T* score < −1.0 or *Z* score ≤ −2.0, osteoporosis: *T* score ≤ −2.5) among T2DM patients stratified by arterial stiffness severity, analyzed by chi‐square test. *p* values are labeled.

Spearman's correlation analysis (Table 2) revealed a significant negative correlation between baPWV and L1–L4 TBS (*ρ* = −0.355, *p* < 0.001). Similarly, *T* scores at the femoral neck (*ρ* = −0.254), total hip (*ρ* = −0.207), and L1–L4 BMD (*ρ* = −0.183) also showed significant inverse correlations with baPWV (all *p* < 0.001). In contrast, no significant associations were observed between baPWV and *Z* scores at any of the measured sites (all *p* > 0.05). Patients was divided based on baPWV levels into normal, elevated, and severe arterial stiffness, with cutoff levels of 1400 and 1800 cm/s. The analysis demonstrated statistically significant differences in osteoporosis prevalence among these groups (*p* < 0.05, Figure 1B). Additionally, the severe arterial stiffness group showed significantly higher rates of low TBS compared to both normal and elevated stiffness groups (both *p* < 0.05, Figure 1A).

**TABLE 2:** Spearman's rank correlation coefficients between baPWV and TBS/BMD.

Variables	Spearman's *ρ*	*p*
FN BMD		
Z score	−0.027	0.788
T score	−0.254	< 0.001
TH BMD		
Z score	−0.061	0.543
T score	−0.207	< 0.001
L1–L4 BMD		
Z score	−0.090	0.373
T score	−0.183	< 0.001
L1–L4 TBS	−0.355	< 0.001

Abbreviations: FN BMD: femoral neck bone mineral density; L1–L4 BMD: lumbar spine 1 to 4 bone mineral density; TBS: trabecular bone score; TH BMD: total hip bone mineral density.

### 3.3 baPWV and L1–L4 TBS Show an Independent Negative Correlation in T2DM Patients, Whereas BMD Demonstrates Only a Bivariate Association

Following logarithmic transformation of baPWV and TBS and standardization of independent variables, incorporate the independent variables exhibiting intergroup differences in Table 1, across the entire population, age (*p* < 0.001), BMI (*p* < 0.001), TPINP (*p* < 0.001), and baPWV (*p* < 0.001) were independent risk factors for L1–L4 TBS, while L1–L4 BMD (*p* < 0.001) and FN BMD (*p* = 0.003) were independent protective factors for L1–L4 TBS (Table 3). Male patients exhibited additional negative associations with DBP (*β* = −0.003, *p* = 0.025) and ALP (*β* = −0.003, *p* = 0.023). Moreover, age and BMI were not significant variables in the female subgroup. We conducted a multi‐model linear regression analysis to examine the relationship between baPWV and L1–L4 TBS (Table 4). In the crude model (Model 1), a 1% increase in baPWV was associated with an average 0.164% decrease in L1–L4 TBS (95% CI: 0.134% to 0.194%, *p* < 0.001). This inverse relationship remained robust after adjusting for age, sex, DBP, BMI, diabetic duration, Hb, BUN, eGFR, UACR, LDL‐C, N‐t OC, TPINP, ALP (Model 3: 95% CI: 0.038% to 0.114%, *p* < 0.001). When analyzed categorically, participants in severe arterial stiffness group (G3) exhibited significantly lower L1–L4 TBS versus the reference group (G1) across all models, with the fully adjusted Model 3 showing *β* = −0.010 (95% CI: −0.018 to −0.001, *p* = 0.034) and a persistent linear trend (*p* for trend = 0.040). The elevated arterial stiffness group (G2) showed attenuated effects that became non‐significant after full adjustment (*p* > 0.05).

**TABLE 3:** Stepwise linear regression analysis of factors associated with L1–L4 TBS among T2DM patients.

	ALL (*n* = 646)				Male (*n* = 444)				Females (*n* = 202)			
	*β* (95% CI)	*p*	Change in *R* ^2^	VIF	*β* (95% CI)	*p*	Change in *R* ^2^	VIF	*β* (95% CI)	*p*	Change in *R* ^2^	VIF
Age	−0.007 (−0.010,−0.004)	< 0.001	0.017	1.863	−0.008 (−0.011,−0.004)	< 0.001	0.029	1.639				
BMI	−0.006 (−0.008,−0.003)	< 0.001	0.020	1.281	−0.008 (−0.011,−0.005)	< 0.001	0.040	1.284				
DBP					−0.003 (−0.006,0.000)	0.025	0.009	1.180	0.005 (0.001,0.009)	0.021	0.014	1.051
TPINP	−0.004 (−0.006,−0.002)	< 0.001	0.015	1.046					−0.005 (−0.009,−0.001)	0.014	0.017	1.049
ALP					−0.003 (−0.005,0.000)	0.023	0.009	1.059				
baPWV	−0.007 (−0.009,−0.004)	< 0.001	0.070	1.476	−0.004 (−0.007,−0.001)	0.019	0.048	1.455	−0.008 (−0.013,−0.004)	< 0.001	0.083	1.403
FN BMD	0.005 (0.002,0.008)	0.003	0.009	1.919					0.009 (0.003,0.015)	0.002	0.028	2.208
UACR									−0.008 (−0.014,‐0.002)	0.006	0.021	1.102
L1–L4 BMD	0.015 (0.012,0.018)	< 0.001	0.332	1.726	0.016 (0.013,0.019)	< 0.001	0.228	1.126	0.013 (0.008,0.019)	< 0.001	0.439	2.165

Abbreviations: ALP: alkaline phosphatase; baPWV: brachial‐ankle pulse wave velocity; BMI: body mass index; CI: confidence interval; DBP: diastolic blood pressure; FN BMD: femoral neck bone mineral density; L1–L4 BMD: lumbar spine 1 to 4 bone mineral density; TPINP: total type I collagen N‐terminal propeptide; UACR: urinary albumin‐to‐creatinine ratio; VIF: variance inflation factor.

**TABLE 4:** Associations between baPWV and L1–L4 TBS in T2DM patients.

	Model 1		Model 2		Model 3	
	*β* (95% CI)	*p*	*β* (95% CI)	*p*	*β* (95% CI)	*p*
baPWV (continuous)	−0.164 (−0.194,−0.134)	< 0.001	−0.087 (−0.120,−0.054)	< 0.001	−0.076 (−0.114,−0.038)	< 0.001
baPWV (group)						
G1 (< 1400)	Ref.		Ref.		Ref.	
G2 (1400–1800)	−0.036 (−0.043,−0.028)	< 0.001	−0.005 (−0.010,0.001)	0.078	−0.001 (−0.007,0.005)	0.757
G3 (> 1800)	−0.013 (−0.019,−0.007)	< 0.001	−0.017 (−0.025,‐0.009)	< 0.001	−0.010 (−0.018,‐0.001)	0.034
*p* for trend	< 0.001		< 0.001		0.040	

*Note:* Model 1: crude model. Model 2: adjusted for age and sex. Model 3: adjusted for age, sex, DBP, BMI, diabetic duration, Hb, BUN, eGFR, UACR, LDL‐C, N‐t OC, TPINP, ALP.

Abbreviations: baPWV: brachial‐ankle pulse wave velocity; CI: confidence interval; Ref: reference.

In the univariable analysis, significant inverse relationships were observed between baPWV and BMD *T* scores at the femoral neck (*β* = −3.50, 95% CI: −4.56 to −2.45, *p* < 0.001), total hip (*β* = −3.00, 95% CI: −4.11 to −1.89, *p* < 0.001), and lumbar spine (*β* = −3.63, 95% CI: −5.11 to −2.15, *p* < 0.001). However, these associations became non‐significant after multivariable adjustment for confounding factors (all *p* > 0.05; Table 5). Conversely, BMD *Z* scores at all measured sites showed no significant associations with baPWV in both univariable and multivariable analyses (all *p* > 0.05). Supplemental univariate and multivariate analyses of the BMD data are provided in the Data S1.

**TABLE 5:** Associations between baPWV and BMD in T2DM patients.

Subgroups	FN BMD					TH BMD				L1–L4 BMD			
	Univar. Reg.			MV Reg.^a^		Univar. Reg.		MV Reg.^b^		Univar. Reg.	MV Reg.^c^		
	*β* (95% CI)		*p*	*β* (95% CI)	*p*	*β* (95% CI)	*p*	*β* (95% CI)	*p*	*β* (95% CI)	*p*	*β* (95% CI)	*p*
*Z* score	*n* = 101	0.84 (−2.57, 4.25)	0.630			−0.52 (−3.94, 2.91)	0.768			−1.99 (−5.97, 1.99)	0.329		
baPWV													
*T* score	*n* = 545	−3.50 (−4.56, −2.45)	< 0.001	−1.06 (−2.36, 0.24)	0.110	−3.00 (−4.11, −1.89)	< 0.001	−0.56 (−1.90, 0.78)	0.415	−3.63 (−5.11, −2.15)	< 0.001	−0.88 (−2.71, 0.94)	0.343
baPWV													

Abbreviations: baPWV: brachial‐ankle pulse wave velocity; CI: confidence interval; FN BMD: femoral neck bone mineral density; L1–L4 BMD: lumbar spine 1 to 4 bone mineral density; MV Reg.: multivariable linear regression coefficients; TH BMD: total hip bone mineral density; Univar. Reg.: univariable linear regression.

^a^ Multivariable linear regression coefficients adjusted for age, sex, DBP, BMI, WC, diabetic duration, ALT, HOMA‐β, Hb, BUN, eGFR, UA, TG, TC, HDL‐c, LDL‐c, N‐t OC, TPINP, β‐CTX, Ca.

^b^ Multivariable linear regression coefficients adjusted for age, sex, DBP, BMI, WC, diabetic duration, ALT, AST, HOMA‐β, Hb, BUN, eGFR, UA, TC, HDL‐c, LDL‐c, N‐t OC, TPINP, β‐CTX, Ca.

^c^ Multivariable linear regression coefficients adjusted for age, sex, DBP, BMI, WC, diabetic duration, ALT, ALP, Hb, BUN, eGFR, UA, TC, HDL‐c, LDL‐c, N‐t OC, TPINP, β‐CTX.

### 3.4 Non‐Linear Correlation Between baPWV and L1–L4 TBS

**FIGURE 2:**
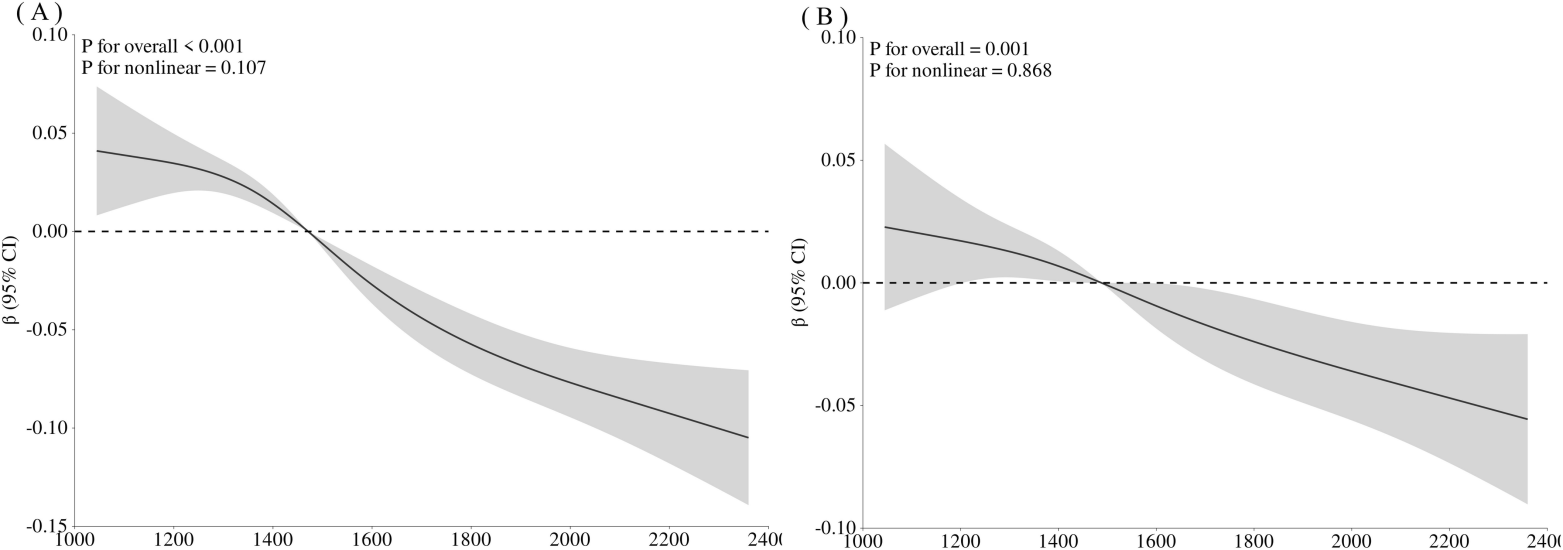
Restricted cubic spline (RCS) curves of L1–L4 TBS with baPWV. (A) Unadjusted model: RCS curve depicting the crude association between baPWV (continuous) and TBS. (B) Adjusted model: RCS curve showing the association between baPWV and TBS after adjustment for age, sex, diastolic blood pressure, body mass index, diabetic duration, hemoglobin, blood urea nitrogen, estimated glomerular filtration rate, urinary albumin‐to‐creatinine ratio, low‐density lipoprotein cholesterol, N‐terminal osteocalcin, total type I collagen N‐terminal propeptide, and alkaline phosphatase.

RCS analysis revealed a significant linear association between baPWV and L1–L4 TBS (Figure 2). The unadjusted model (Figure 2A) showed a strong overall inverse relationship (*p* for overall < 0.001). After full adjustment for age, sex, DBP, BMI, Diabetic duration, Hb, BUN, eGFR, UACR, LDL‐C, N‐t OC, TPINP, ALP (Figure 2B), the overall association remained significant (*p* for overall = 0.001), with the curve exhibiting an approximately linear pattern (*p* for nonlinear = 0.868).

### 3.5 ROC Curves for baPWV Prediction of Low TBS

**FIGURE 3:**
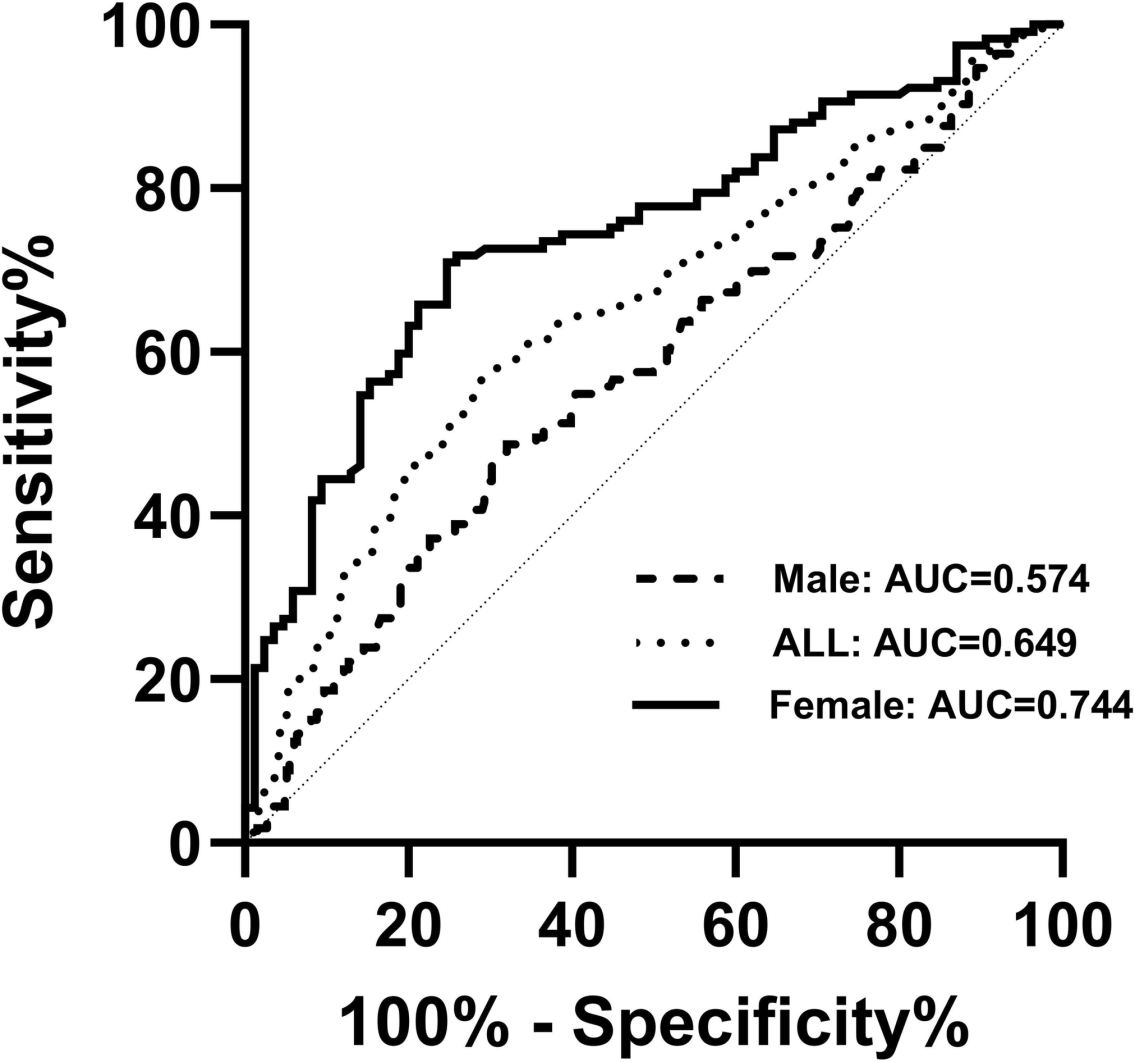
Receiver operating characteristic (ROC) curve of baPWV. ROC curves demonstrate the discriminative ability of brachial‐ankle pulse wave velocity (baPWV) for identifying degraded bone microarchitecture, defined as trabecular bone score (TBS) < 1.31. The diagonal line represents the reference line of no discriminative ability (AUC = 0.5).

TBS < 1.310 is defined as the low TBS. The outcomes are presented in Figure 3 and Table 6. Compared to the overall and male population, the area under the curve (AUC) is maximal in female, at 0.744 (95% CI = 0.677–0.812, *p* < 0.001), with an optimal cutoff point of 1506. The Youden's index is 0.462, sensitivity is 0.709, and specificity is 0.753.

**TABLE 6:** The value of baPWV in forecasting the low TBS in patients with T2DM.

	AUC (95% CI)	Youden's index	Sensitivity	Specificity	*p*
baPWV (All)	0.649 (0.604–0.695)	0.277	0.587	0.69	< 0.001
baPWV (Male)	0.574 (0.511–0.637)	0.167	0.487	0.68	0.019
baPWV (Female)	0.744 (0.677–0.812)	0.462	0.709	0.753	< 0.001

Abbreviations: AUC: area under the curve; baPWV: brachial‐ankle pulse wave velocity.

## 4 Discussion

The present study investigated factors associated with L1–L4 TBS in patients with T2DM. The results showed that age, sex, BMI, TPINP, ALP, DBP, UACR, FN BMD, L1–L4 BMD, and baPWV independently influenced L1–L4 TBS. This study is the first to identify baPWV as an independent risk factor for L1–L4 TBS in patients with T2DM, with a consistent linear negative correlation observed across gender subgroups. This indicates that in clinical practice, for patients with T2DM complicated by arterial stiffness, it is essential to focus not only on cardiovascular risk assessment but also pay particular attention to skeletal health issues.

Bone microstructure degradation may be one of the potential mechanisms that lead to normal BMD but increased fracture risk in patients with T2DM [14]. As a new index for assessing bone microarchitecture, the TBS effectively compensates for the shortcomings of BMD in forecasting fracture risk. Previous studies have proposed that BMI may affect TBS levels [14]. But the conclusions remain inconsistent across studies. A pharmacodynamic longitudinal study [15] showed that TBS increased slightly with decreasing BMI. Qi et al. [16] studied 243 postmenopausal women and did not find any differences in TBS levels among normal, overweight, and obese populations. A study on American adolescents showed slight positive connection between TBS and BMI [17]. Nevertheless, Our research findings indicate a negative correlation between the two in patients with T2DM, implying that obesity could act as an independent risk factor for the low TBS. However, recent findings suggest that soft tissue thickness at the measurement site can influence TBS measurements, and that different individual morphologies can lead to heterogeneous distribution of soft tissue thickness at the same BMI levels [18]. A research study found that in the face of individuals with significantly thickened soft tissue, DXA absorbs more radiation during data collecting, resulting in lower “raw” TBS results [19].

Patients with diabetes have reduced levels of bone turnover markers, such as PINP, OCN, PTH, bone‐specific phosphatase, and CTX [20], and these bone metabolism markers have been reported to correlate with BMD [21] and TBS. Ubago‐Guisado et al. [7] indicated that TBS correlates with PINP and CTX in individuals with T2DM. In contrast to that study, we discovered that PINP independently affected TBS, but no significant link existed between CTX and TBS. This may pertain to the variability of data or testing instruments. In the male population, L1–L4 TBS was independently and negatively correlated with ALP. Pini et al. [22] also found this relationship in patients with diffuse idiopathic osteomalacia but found no correlation between the two in patients with X‐linked hypophosphatemia [23].

baPWV, a simple and noninvasive measure of arterial stiffness, is among the strongest predictors of mortality and cardiovascular disease independent of conventional vascular risk factors [24]. To date, few studies have systematically investigated the relationship between baPWV and TBS. Yano et al. [25] were the first to find a correlation between baPWV and TBS in a study of patients with adrenal tumors. But they did not perform an in‐depth analysis. We first found a substantial negative correlation between baPWV and TBS in patients with T2DM which existed in different gender subgroups. We propose that arterial stiffness was an independent risk factor for low TBS. However, one study assessed bone microstructure by high‐resolution peripheral quantitative computed tomography (HR‐pQCT) but failed to demonstrate an independent association between vascular function and bone microstructure [26]. This may relate to differences in the population and measurement techniques. Firstly, the aforementioned study was performed on the general population, while our focus was specifically on patients with T2DM. These patients exhibit increased cortical porosity and larger holes in the trabecular network, suggesting that their bone microarchitecture is more vulnerable to deterioration [27]. Secondly, HR‐pQCT evaluates peripheral bone, mainly composed of cortical bone, while TBS examines the lumbar spine, which is rich in cancellous bone [28]. Due to its higher metabolic turnover and abundant blood supply, cancellous bone may undergo alterations prior to cortical bone in the context of diabetes [29]. Moreover, A review indicates discordant changes in trabecular bone microstructure between HR‐pQCT and TBS in T2DM [30].

The decrease in TBS is accompanied by an increase in arterial stiffness, which may be mediated through two distinct pathophysiological mechanisms. Firstly, nutrient arteries are the primary source of blood supply to the bones. In lengthy bones, blood is delivered to the bones in an inside‐out pattern. Senescence and vascular pathology may result in vascular stiffness or blockage, subsequently altering the pattern of blood flow. The outside‐in supply pattern results in inadequate bone perfusion, thereby impacting bone density and microstructure [31]. Moreover, patients with T2DM exhibit worse vascular conditions, with vascular lesions occurring earlier and exerting a more adverse impact on bone perfusion. Secondly, the elevated blood glucose environment accelerates the pile‐up of advanced glycation end‐products (AGEs) [32]. Abnormal cross‐linking between AGEs and key molecules in the extracellular matrix might result in decreased vascular elasticity, heightened vascular stiffness, and worsening hypoxia [31, 33]. Hypoxia can increase the production of proinflammatory cytokines and reactive oxygen species (ROS), thereby affecting bone metabolism [34, 35]. On the one hand, the elevated expression of pro‐inflammatory cytokines, such as tumor necrosis factor‐alpha (TNF‐α) is associated with a marked increase in osteoblast apoptosis [36], On the other hand, oxidative stress may disrupt the osteogenic differentiation of mesenchymal stem cells (MSCs) [37]. Conversely, the overproduction of ROS might accelerate the formation of AGEs, so exacerbating the detrimental effects of proinflammatory cytokine and ROS on bone metabolism, thereby forming a vicious cycle [38, 39].

Gender disparities exist in the occurrence of osteoporosis. With increasing age and a decreasing gonadal function, sex steroid hormones are ineffective in promoting bone remodeling and enhancing bone strength, thus accelerating the onset of osteoporosis [40]. Bone loss occurs earlier in women than in men due to the different actions of testosterone and estradiol on the periosteum [41], and studies have shown that the action of the androgen receptor pathway maintains or increases the trabecular number and inhibits trabecular resorption [42, 43]. Our study included a subgroup analysis based on gender. Both male and female patients demonstrated that baPWV was an independent factor influencing L1–L4 TBS and its predictive capacity for low TBS being superior in females. Therefore, it is justifiable to say that the correlation between the two is more pronounced in women.

The findings of current research regarding the association between baPWV and BMD are contradictory. Dong Ke Liang et al. [44] determined no significant association, whereas Xue Song Li et al. [45] identified a correlation among hypertensive males. Importantly, no research has investigated the connection between baPWV and BMD in patients with T2DM. Our study revealed that although a bivariate correlation existed between baPWV and BMD, this correlation was negated after multifactorial adjustment. Conversely, baPWV exhibited an independent negative correlation with TBS. This may be due to the phenomenon that, in patients with T2DM, BMD may remain normal or even elevated, while the TBS demonstrates a significant decline [4, 7].

This study addresses the gap in the literature on the correlation between baPWV and TBS in patients with T2DM, revealing that baPWV has a predictive value for low TBS, which is potentially valuable for early bone microarchitectural assessment and fracture risk prevention in patients with T2DM undergoing screening for arterial stiffness.

This study has some limitations. First, the cross‐sectional study design limited the assessment of causal relationships between factors associated with L1–L4 TBS. Second, this study lacked healthy population controls to examine the relationship between baPWV and L1–L4 TBS in the general population and did not provide insight into whether diabetes exacerbates the effect of arterial stiffness on TBS. Furthermore, the sample size presented in this article is inadequate, and the sample source is very homogeneous. Future research should implement a longitudinal study design to clarify the causal linkages among relevant factors, concentrating on investigating the experimental pathways between arterial stiffness and bone microstructural degeneration to reveal the physiological foundation of their interactions.

## 5 Conclusion

In summary, this study showed that baPWV was independently and negatively correlated with L1–L4 TBS, which can predict the occurrence of low TBS, with a more marked prediction capacity in women. Therefore, the present study tentatively suggests that patients with T2DM and arterial stiffness may be at a higher risk of bone microarchitectural degeneration. However, in‐depth validation in larger, multicenter, longitudinal cohort studies is required.

## Author Contributions

Yangting Wang: Evaluated all the image data and participated in drafting the manuscript. Xiaoting Chen: Evaluated all the image data and participated in drafting the manuscript. Jinjian Xu: Formal analysis, analyzed data. Qifeng Ying: Contributed to the discussion and revision of the manuscript. Lizhi Liang: Contributed to the collection of data. Xiaohong Wu: Supervised this project, obtained funding, and finalized the manuscript.

## Funding

This work was supported by the Joint Funds of the Zhejiang Provincial Natural Science Foundation of China, LHDMZ23H070001, Zhejiang Province Science and Technology Plan Project, 2021R52022, and Zhejiang province health innovative talents project, 2021‐CXRC07‐01.

## Ethics Statement

This research was performed in compliance with the Declaration of Helsinki. The Ethics Committee of Zhejiang Provincial People's Hospital sanctioned the study, assigning approval number ZJPPHEC 2024O (275). This study utilized fully anonymized data containing no personally identifiable images, with an informed consent waiver approved by the Ethics Committee.

## Conflicts of Interest

The authors declare no conflicts of interest.

## Supporting information


**Data S1:** jdb70181‐sup‐0001‐Supinfo.pdf.

## Data Availability

The data that support the findings of this study are available on request from the corresponding author. The data are not publicly available due to privacy or ethical restrictions.
