# Supplementary material for: Association Between Arterial Stiffness and Bone Microarchitectural Deterioration in Type 2 Diabetes: A Cross‐Sectional Study
Source: J Diabetes. 2025 Dec 10;17(12):e70181. doi: 10.1111/1753-0407.70181 (PMC12695132; doi:10.1111/1753-0407.70181)
Supplement: Supplementary file 1 — Data S1: jdb70181‐sup‐0001‐Supinfo.pdf. [file JDB-17-e70181-s001.pdf]

Vascular stiffness is an independent risk factor for low trabecular bone score in T2DM patients: A cross-sectional study

Authors' full names and institutions

Yangting Wang[1#], Xiaoting Chen[2#], Jinjian Xu[2], Qifeng Ying[3], Lizhi Liang[3], Xiaohong Wu[2*]

[1] Hangzhou Normal University, Hangzhou 311121, China.

[2] Geriatric Medicine Center, Key Laboratory of Endocrine Gland Diseases of Zhejiang Province, Department of Endocrinology, Zhejiang Provincial People's Hospital (Affiliated People's Hospital, Hangzhou Medical College), Hangzhou 310014, Zhejiang, China

[3] Department of Osteoporosis Diagnosis and Treatment Center, Zhejiang Provincial People's Hospital (Affiliated People's Hospital, Hangzhou Medical College), Hangzhou 310014, China.

Authors' e-mail addresses:

Yangting Wang, wwyt0220@163.com

Xiaoting Chen, chenxiaoting@hmc.edu.cn

Jinjian Xu, xujj56@mail2.sysu.edu.cn

Qifeng Ying, yingqifeng@hmc.edu.cn

Lizhi Liang, lianglizhi@hmc.edu.cn

Xiaohong Wu, drxhwu@njmu.edu.cn

[#] These authors contributed equally to this work.

*** Correspondence to** Xiaohong Wu, Email: drxhwu@njmu.edu.cn. ORCiD: 0000-0001-5984-3342

Reprint requests should be addressed to Xiaohong Wu, Zhejiang Provincial People's Hospital, 158 Shangtang Rd, Hangzhou 310014, Zhejiang, China.

Supporting Material（Expanded Version of Table 5）

Table 1 Linear regression of baPWV and FN BMD Z-score in T2DM patients

Variables	Univar. Reg.					MV Reg.				
	β	S.E	t	*P*	β (95%CI)	β	S.E	t	*P*	β (95%CI)
Sex										
Male					0.00 (Reference)					0.00 (Reference)
Female	0.63	0.24	2.59	**0.011**	0.63 (0.15 ~ 1.11)	0.63	0.26	2.39	**0.019**	0.63 (0.11 ~ 1.14)
Age	0.01	0.02	0.81	0.419	0.01 (-0.02 ~ 0.04)					
DBP	0.01	0.01	1.13	0.263	0.01 (-0.01 ~ 0.03)					
SBP	0.00	0.01	0.06	0.952	0.00 (-0.01 ~ 0.01)					
BMI	0.03	0.03	1.28	0.205	0.03 (-0.02 ~ 0.08)					
WC	0.02	0.01	1.58	0.117	0.02 (-0.00 ~ 0.04)					
Diabetic duration	0.00	0.00	0.13	0.895	0.00 (-0.00 ~ 0.01)					
HbA1c	-0.05	0.04	-1.11	0.271	-0.05 (-0.13 ~ 0.04)					
Hb	-0.00	0.01	-0.17	0.863	-0.00 (-0.01 ~ 0.01)					
ALT	-0.00	0.00	-0.96	0.342	-0.00 (-0.01 ~ 0.00)					
AST	0.00	0.00	0.41	0.686	0.00 (-0.00 ~ 0.01)					
ALP	-0.00	0.00	-0.84	0.401	-0.00 (-0.01 ~					

Variables	Univar. Reg.					MV Reg.				
	β	S.E	t	*P*	β (95%CI)	β	S.E	t	*P*	β (95%CI)
					0.01)					
BUN	-0.03	0.06	-0.53	0.600	-0.03 (-0.16 ~ 0.09)					
Cr	-0.01	0.00	-2.06	**0.042**	-0.01 (-0.02 ~ -0.01)	-0.00	0.00	-0.98	0.329	-0.00 (-0.01 ~ 0.00)
eGFR	0.00	0.01	0.74	0.461	0.00 (-0.01 ~ 0.02)					
UACR	0.00	0.00	0.02	0.985	0.00 (-0.00 ~ 0.00)					
UA	0.00	0.00	1.59	0.115	0.00 (-0.00 ~ 0.00)					
TG	0.04	0.03	1.25	0.215	0.04 (-0.02 ~ 0.10)					
TC	0.12	0.07	1.55	0.125	0.12 (-0.03 ~ 0.26)					
HDLc	0.08	0.55	0.14	0.891	0.08 (-1.01 ~ 1.16)					
LDLc	0.10	0.14	0.71	0.481	0.10 (-0.17 ~ 0.37)					
HOMA-IR	0.01	0.01	1.28	0.205	0.01 (-0.01 ~ 0.03)					
HOMA-β	0.00	0.00	0.59	0.559	0.00 (-0.00 ~ 0.00)					
ABL	2.71	1.12	2.41	**0.018**	2.71 (0.50 ~ 4.91)	3.18	1.09	2.91	**0.004**	3.18 (1.04 ~ 5.32)
baPWV	0.84	1.74	0.48	0.630	0.84 (-2.57 ~ 4.25)					
PTH	0.01	0.01	1.08	0.282	0.01 (-0.01 ~ 0.02)					
N-t OC	-0.02	0.02	-0.99	0.325	-0.02 (-0.07 ~ 0.02)					
TPINP	-0.00	0.01	-0.80	0.426	-0.00 (-0.02 ~ 0.01)					
β-CTX	-0.00	0.00	-1.21	0.230	-0.00 (-0.00 ~ 0.00)					
25 (OH)D	0.01	0.02	0.41	0.681	0.01 (-0.03 ~ 0.05)					

Variables	Univar. Reg.					MV Reg.				
	β	S.E	t	*P*	β (95%CI)	β	S.E	t	*P*	β (95%CI)
Ca	1.13	0.95	1.19	0.236	1.13 (-0.73 ~ 2.98)					
P	0.86	0.44	1.95	0.055	0.86 (-0.01 ~ 1.73)					

Table 2 Linear regression of baPWV and TH BMD Z-score in T2DM patients

Variables	Univar. Reg.					MV Reg.				
	β	S.E	t	*P*	β (95%CI)	β	S.E	t	*P*	β (95%CI)
Sex										
Male					0.00 (Reference)					0.00 (Reference)
Female	0.93	0.23	3.99	**<.001**	0.93 (0.47 ~ 1.39)	0.86	0.26	3.25	**0.002**	0.86 (0.34 ~ 1.38)
Age	-0.00	0.02	-0.32	0.752	-0.00 (-0.03 ~ 0.03)					
DBP	0.01	0.01	0.69	0.492	0.01 (-0.01 ~ 0.02)					
SBP	0.00	0.01	0.12	0.903	0.00 (-0.01 ~ 0.01)					
BMI	0.06	0.03	2.38	**0.019**	0.06 (0.01 ~ 0.11)	-0.02	0.04	-0.53	0.598	-0.02 (-0.11 ~ 0.06)
WC	0.02	0.01	2.12	**0.036**	0.02 (0.01 ~ 0.04)	0.03	0.02	1.92	0.058	0.03 (-0.00 ~ 0.07)
Diabetic duration	0.00	0.00	0.14	0.891	0.00 (-0.00 ~ 0.01)					
HbA1c	-0.08	0.04	-1.95	0.054	-0.08 (-0.17 ~ 0.00)					
Hb	-0.00	0.01	-0.30	0.767	-0.00 (-0.02 ~ 0.01)					
ALT	0.00	0.00	0.30	0.762	0.00 (-0.00 ~ 0.01)					
AST	0.01	0.00	1.73	0.087	0.01 (-0.00 ~ 0.01)					
ALP	-0.01	0.00	-1.32	0.191	-0.01 (-0.02 ~ 0.00)					
BUN	-0.07	0.06	-1.15	0.251	-0.07 (-0.20 ~					

Variables	Univar. Reg.					MV Reg.				
	β	S.E	t	*P*	β (95%CI)	β	S.E	t	*P*	β (95%CI)
					0.05)					
Cr	-0.01	0.00	-2.38	**0.019**	-0.01 (-0.02 ~ -0.01)	-0.01	0.00	-1.36	0.177	-0.01 (-0.02 ~ 0.00)
eGFR	0.01	0.01	1.18	0.242	0.01 (-0.00 ~ 0.02)					
UACR	0.00	0.00	0.02	0.980	0.00 (-0.00 ~ 0.00)					
UA	0.00	0.00	1.63	0.106	0.00 (-0.00 ~ 0.00)					
TG	0.04	0.03	1.18	0.242	0.04 (-0.02 ~ 0.09)					
TC	0.09	0.08	1.14	0.256	0.09 (-0.06 ~ 0.23)					
HDLc	0.22	0.56	0.40	0.687	0.22 (-0.86 ~ 1.31)					
LDLc	0.04	0.14	0.30	0.766	0.04 (-0.23 ~ 0.32)					
HOMA-IR	0.01	0.01	1.06	0.292	0.01 (-0.01 ~ 0.03)					
HOMA-β	-0.00	0.00	-0.09	0.931	-0.00 (-0.00 ~ 0.00)					
ABL	2.07	1.14	1.82	0.072	2.07 (-0.16 ~ 4.31)					
baPWV	-0.52	1.75	-0.30	0.768	-0.52 (-3.94 ~ 2.91)					
PTH	0.01	0.01	1.00	0.319	0.01 (-0.01 ~ 0.02)					
N-t OC	-0.04	0.02	-1.64	0.105	-0.04 (-0.09 ~ 0.01)					
TPINP	-0.01	0.01	-1.17	0.247	-0.01 (-0.02 ~ 0.00)					
β-CTX	-0.00	0.00	-1.54	0.127	-0.00 (-0.00 ~ 0.00)					
25 (OH)D	0.01	0.02	0.58	0.561	0.01 (-0.03 ~ 0.05)					
Ca	1.58	0.95	1.67	0.098	1.58 (-0.27 ~ 3.44)					

Variables	Univar. Reg.					MV Reg.				
	β	S.E	t	*P*	β (95%CI)	β	S.E	t	*P*	β (95%CI)
P	0.71	0.45	1.57	0.119	0.71 (-0.17 ~ 1.59)					

Table 3 Linear regression of baPWV and L1-L4 BMD Z-score in T2DM patients

Variables	Univar. Reg.					MV Reg.				
	β	S.E	t	*P*	β (95%CI)	β	S.E	t	*P*	β (95%CI)
Sex										
Male					0.00 (Reference)					0.00 (Reference)
Female	0.86	0.28	3.05	**0.003**	0.86 (0.31 ~ 1.41)	0.83	0.29	2.83	**0.006**	0.83 (0.26 ~ 1.41)
Age	-0.02	0.02	-1.19	0.238	-0.02 (-0.06 ~ 0.01)					
DBP	0.01	0.01	1.34	0.183	0.01 (-0.01 ~ 0.03)					
SBP	0.01	0.01	1.40	0.166	0.01 (-0.00 ~ 0.03)					
BMI	0.06	0.03	2.18	**0.032**	0.06 (0.01 ~ 0.12)	-0.02	0.05	-0.48	0.636	-0.02 (-0.12 ~ 0.08)
WC	0.03	0.01	2.15	**0.034**	0.03 (0.01 ~ 0.05)	0.03	0.02	1.53	0.131	0.03 (-0.01 ~ 0.07)
Diabetic duration	0.00	0.00	0.73	0.466	0.00 (-0.00 ~ 0.01)					
HbA1c	-0.03	0.05	-0.58	0.562	-0.03 (-0.13 ~ 0.07)					
Hb	-0.01	0.01	-1.16	0.251	-0.01 (-0.03 ~ 0.01)					
ALT	0.00	0.00	0.07	0.944	0.00 (-0.01 ~ 0.01)					
AST	0.00	0.00	0.96	0.338	0.00 (-0.00 ~ 0.01)					
ALP	-0.00	0.01	-0.14	0.893	-0.00 (-0.01 ~ 0.01)					
BUN	0.02	0.08	0.27	0.788	0.02 (-0.13 ~ 0.17)					
Cr	-0.00	0.01	-0.56	0.576	-0.00 (-0.01 ~					

Variables	Univar. Reg.					MV Reg.				
	β	S.E	t	*P*	β (95%CI)	β	S.E	t	*P*	β (95%CI)
					0.01)					
eGFR	-0.00	0.01	-0.00	0.996	-0.00 (-0.01 ~ 0.01)					
UACR	0.00	0.00	1.50	0.136	0.00 (-0.00 ~ 0.00)					
UA	0.00	0.00	1.47	0.145	0.00 (-0.00 ~ 0.00)					
TG	0.10	0.03	2.98	**0.004**	0.10 (0.03 ~ 0.17)	0.05	0.05	1.17	0.245	0.05 (-0.04 ~ 0.14)
TC	0.23	0.09	2.70	**0.008**	0.23 (0.06 ~ 0.40)	0.08	0.11	0.73	0.465	0.08 (-0.14 ~ 0.31)
HDLc	0.56	0.65	0.87	0.389	0.56 (-0.71 ~ 1.83)					
LDLc	0.14	0.16	0.83	0.408	0.14 (-0.18 ~ 0.46)					
HOMA-IR	0.01	0.01	0.91	0.362	0.01 (-0.01 ~ 0.03)					
HOMA-β	-0.00	0.00	-0.07	0.948	-0.00 (-0.00 ~ 0.00)					
ABL	2.24	1.34	1.68	0.096	2.24 (-0.37 ~ 4.86)					
baPWV	-1.99	2.03	-0.98	0.329	-1.99 (-5.97 ~ 1.99)					
PTH	0.01	0.01	0.82	0.412	0.01 (-0.01 ~ 0.02)					
N-t OC	-0.04	0.03	-1.53	0.129	-0.04 (-0.10 ~ 0.01)					
TPINP	-0.01	0.01	-1.51	0.135	-0.01 (-0.02 ~ 0.00)					
β-CTX	-0.00	0.00	-1.57	0.119	-0.00 (-0.00 ~ 0.00)					
25 (OH)D	0.03	0.02	1.25	0.214	0.03 (-0.02 ~ 0.08)					
Ca	0.75	1.10	0.68	0.501	0.75 (-1.42 ~ 2.91)					
P	0.24	0.52	0.47	0.640	0.24 (-0.77 ~ 1.26)					

Table 4 Linear regression of baPWV and FN BMD T-score in T2DM patients

Variables	Univar. Reg.					MV Reg.				
	β	S.E	t	*P*	β (95%CI)	β	S.E	t	*P*	β (95%CI)
Sex										
Male					0.00 (Reference)					0.00 (Reference)
Female	-0.55	0.09	-5.80	**<.001**	-0.55 (-0.73 ~ -0.36)	-0.18	0.13	-1.39	0.164	-0.18 (-0.42 ~ 0.07)
Age	-0.04	0.00	-11.24	**<.001**	-0.04 (-0.05 ~ -0.03)	-0.03	0.01	-5.01	**<.001**	-0.03 (-0.04 ~ -0.02)
DBP	0.02	0.00	3.78	**<.001**	0.02 (0.01 ~ 0.02)	-0.00	0.00	-0.94	0.345	-0.00 (-0.01 ~ 0.00)
SBP	-0.00	0.00	-0.16	0.874	-0.00 (-0.01 ~ 0.00)					
BMI	0.10	0.01	9.11	**<.001**	0.10 (0.08 ~ 0.12)	0.03	0.02	2.16	**0.032**	0.03 (0.01 ~ 0.06)
WC	0.02	0.00	7.24	**<.001**	0.02 (0.02 ~ 0.03)	0.01	0.00	2.79	**0.005**	0.01 (0.01 ~ 0.02)
Diabetic duration	-0.01	0.00	-3.96	**<.001**	-0.01 (-0.99 ~ -0.01)	0.00	0.00	0.94	0.346	0.00 (-0.00 ~ 0.00)
HbA1c	0.01	0.02	0.51	0.611	0.01 (-0.03 ~ 0.05)					
Hb	0.01	0.00	4.99	**<.001**	0.01 (0.01 ~ 0.02)	0.00	0.00	0.25	0.799	0.00 (-0.01 ~ 0.01)
ALT	0.01	0.00	4.05	**<.001**	0.01 (0.01 ~ 0.01)	0.00	0.00	0.04	0.967	0.00 (-0.00 ~ 0.00)
AST	0.00	0.00	1.82	0.069	0.00 (-0.00 ~ 0.01)					
ALP	-0.00	0.00	-1.77	0.077	-0.00 (-0.01 ~ 0.00)					
BUN	-0.09	0.02	-3.97	**<.001**	-0.09 (-0.14 ~ -0.05)	-0.03	0.03	-1.20	0.229	-0.03 (-0.09 ~ 0.02)
Cr	0.00	0.00	0.43	0.670	0.00 (-0.00 ~ 0.00)					
eGFR	0.01	0.00	5.65	**<.001**	0.01 (0.01 ~ 0.02)	-0.00	0.00	-0.65	0.514	-0.00 (-0.01 ~ 0.00)
UACR	-0.00	0.00	-1.13	0.260	-0.00 (-0.00 ~ 0.00)					

Variables	Univar. Reg.					MV Reg.				
	β	S.E	t	*P*	β (95%CI)	β	S.E	t	*P*	β (95%CI)
UA	0.01	0.00	4.21	**<.001**	0.01 (0.01 ~ 0.01)	0.00	0.00	0.91	0.361	0.00 (-0.00 ~ 0.00)
TG	0.04	0.02	2.21	**0.028**	0.04 (0.01 ~ 0.08)	-0.01	0.03	-0.18	0.859	-0.01 (-0.06 ~ 0.05)
TC	0.10	0.04	2.94	**0.003**	0.10 (0.03 ~ 0.17)	0.00	0.07	0.05	0.956	0.00 (-0.13 ~ 0.14)
HDLc	-0.60	0.18	-3.40	**<.001**	-0.60 (-0.95 ~ -0.25)	0.08	0.20	0.42	0.678	0.08 (-0.31 ~ 0.47)
LDLc	0.17	0.05	3.15	**0.002**	0.17 (0.06 ~ 0.28)	0.04	0.09	0.40	0.688	0.04 (-0.14 ~ 0.21)
HOMA-IR	-0.00	0.00	-1.76	0.080	-0.00 (-0.01 ~ 0.00)					
HOMA-β	-0.01	0.00	-2.88	**0.004**	-0.01 (-0.99 ~ -0.01)	-0.01	0.00	-2.40	**0.017**	-0.01 (-0.99 ~ -0.01)
ABL	-0.60	0.41	-1.45	0.147	-0.60 (-1.40 ~ 0.21)					
baPWV	-3.50	0.54	-6.49	**<.001**	-3.50 (-4.56 ~ -2.45)	-1.06	0.66	-1.60	0.110	-1.06 (-2.36 ~ 0.24)
PTH	-0.00	0.00	-1.37	0.171	-0.00 (-0.01 ~ 0.00)					
N-t OC	-0.04	0.01	-4.52	**<.001**	-0.04 (-0.06 ~ -0.02)	-0.00	0.01	-0.21	0.837	-0.00 (-0.03 ~ 0.03)
TPINP	-0.01	0.00	-2.36	**0.018**	-0.01 (-0.99 ~ -0.01)	-0.00	0.00	-0.25	0.801	-0.00 (-0.01 ~ 0.01)
β-CTX	-0.01	0.00	-3.02	**0.003**	-0.01 (-0.99 ~ -0.01)	-0.01	0.00	-2.42	**0.016**	-0.01 (-0.99 ~ -0.01)
25 (OH)D	-0.01	0.01	-0.99	0.322	-0.01 (-0.02 ~ 0.01)					
Ca	0.95	0.40	2.36	**0.019**	0.95 (0.16 ~ 1.73)	0.41	0.40	1.02	0.308	0.41 (-0.37 ~ 1.19)
P	0.00	0.00	0.72	0.473	0.00 (-0.00 ~ 0.01)					

Table 5 Linear regression of baPWV and TH BMD T-score in T2DM patients

Variables	Univar. Reg.					MV Reg.				
	β	S.E	t	*P*	β (95%CI)	β	S.E	t	*P*	β (95%CI)

Variables	Univar. Reg.					MV Reg.				
	β	S.E	t	*P*	β (95%CI)	β	S.E	t	*P*	β (95%CI)
Sex										
Male					0.00 (Reference)					0.00 (Reference)
Female	-0.38	0.10	-3.83	**<.001**	-0.38 (-0.58 ~ -0.19)	-0.02	0.13	-0.16	0.873	-0.02 (-0.28 ~ 0.24)
Age	-0.03	0.00	-9.33	**<.001**	-0.03 (-0.04 ~ -0.03)	-0.03	0.01	-4.26	**<.001**	-0.03 (-0.04 ~ -0.01)
DBP	0.01	0.00	2.96	**0.003**	0.01 (0.01 ~ 0.02)	-0.01	0.00	-1.82	0.070	-0.01 (-0.02 ~ 0.00)
SBP	0.00	0.00	0.20	0.838	0.00 (-0.00 ~ 0.01)					
BMI	0.12	0.01	10.89	**<.001**	0.12 (0.10 ~ 0.14)	0.08	0.02	4.90	**<.001**	0.08 (0.05 ~ 0.12)
WC	0.02	0.00	7.73	**<.001**	0.02 (0.02 ~ 0.03)	0.00	0.01	0.54	0.588	0.00 (-0.01 ~ 0.01)
Diabetic duration	-0.01	0.00	-3.61	**<.001**	-0.01 (-0.99 ~ -0.01)	0.00	0.00	0.91	0.364	0.00 (-0.00 ~ 0.00)
HbA1c	0.02	0.02	0.90	0.369	0.02 (-0.02 ~ 0.06)					
Hb	0.01	0.00	4.64	**<.001**	0.01 (0.01 ~ 0.02)	0.00	0.00	0.79	0.431	0.00 (-0.00 ~ 0.01)
ALT	0.01	0.00	4.38	**<.001**	0.01 (0.01 ~ 0.01)	0.01	0.00	2.18	**0.030**	0.01 (0.01 ~ 0.01)
AST	0.01	0.00	2.16	**0.031**	0.01 (0.01 ~ 0.01)	-0.01	0.00	-2.43	**0.016**	-0.01 (-0.02 ~ -0.01)
ALP	-0.00	0.00	-1.84	0.066	-0.00 (-0.01 ~ 0.00)					
BUN	-0.10	0.02	-4.26	**<.001**	-0.10 (-0.15 ~ -0.05)	-0.05	0.03	-1.83	0.067	-0.05 (-0.11 ~ 0.00)
Cr	-0.00	0.00	-0.05	0.956	-0.00 (-0.00 ~ 0.00)					
eGFR	0.01	0.00	4.92	**<.001**	0.01 (0.01 ~ 0.02)	-0.01	0.00	-1.77	0.077	-0.01 (-0.01 ~ 0.00)
UACR	-0.00	0.00	-1.00	0.315	-0.00 (-0.00 ~ 0.00)					
UA	0.01	0.00	3.30	**0.001**	0.01 (0.01 ~ 0.01)	0.00	0.00	0.00	0.999	0.00 (-0.00 ~ 0.00)

Variables	Univar. Reg.					MV Reg.				
	β	S.E	t	*P*	β (95%CI)	β	S.E	t	*P*	β (95%CI)
TG	0.03	0.02	1.75	0.080	0.03 (-0.00 ~ 0.07)					
TC	0.09	0.04	2.51	**0.013**	0.09 (0.02 ~ 0.16)	0.03	0.05	0.52	0.606	0.03 (-0.08 ~ 0.13)
HDLc	-0.51	0.18	-2.79	**0.006**	-0.51 (-0.88 ~ -0.15)	0.20	0.19	1.03	0.305	0.20 (-0.18 ~ 0.58)
LDLc	0.16	0.06	2.83	**0.005**	0.16 (0.05 ~ 0.27)	-0.01	0.08	-0.17	0.868	-0.01 (-0.17 ~ 0.14)
HOMA-IR	-0.00	0.00	-1.20	0.232	-0.00 (-0.01 ~ 0.00)					
HOMA-β	-0.01	0.00	-2.00	**0.046**	-0.01 (-0.99 ~ -0.01)	-0.00	0.00	-1.59	0.114	-0.00 (-0.00 ~ 0.00)
ABL	-0.60	0.43	-1.40	0.161	-0.60 (-1.44 ~ 0.24)					
baPWV	-3.00	0.57	-5.28	**<.001**	-3.00 (-4.11 ~ -1.89)	-0.56	0.68	-0.82	0.415	-0.56 (-1.90 ~ 0.78)
PTH	-0.00	0.00	-1.01	0.312	-0.00 (-0.01 ~ 0.00)					
N-t OC	-0.05	0.01	-5.28	**<.001**	-0.05 (-0.07 ~ -0.03)	-0.02	0.02	-1.29	0.199	-0.02 (-0.05 ~ 0.01)
TPINP	-0.01	0.00	-2.76	**0.006**	-0.01 (-0.01 ~ -0.01)	-0.00	0.00	-0.20	0.841	-0.00 (-0.01 ~ 0.01)
β-CTX	-0.01	0.00	-3.42	**<.001**	-0.01 (-0.99 ~ -0.01)	-0.00	0.00	-1.76	0.080	-0.00 (-0.00 ~ 0.00)
25 (OH)D	-0.01	0.01	-1.01	0.312	-0.01 (-0.02 ~ 0.01)					
Ca	0.83	0.41	2.00	**0.046**	0.83 (0.02 ~ 1.64)	0.37	0.41	0.90	0.366	0.37 (-0.43 ~ 1.17)
P	0.00	0.00	0.48	0.630	0.00 (-0.00 ~ 0.01)					

Table 6. Linear regression of baPWV and L1-L4 BMD T-score in T2DM patients

Variables	Univar. Reg.					MV Reg.				
	β	S.E	t	*P*	β (95%CI)	β	S.E	t	*P*	β (95%CI)
Sex										
Male					0.00					0.00

Variables	Univar. Reg.					MV Reg.				
	β	S.E	t	*P*	β (95%CI)	β	S.E	t	*P*	β (95%CI)
					(Reference)					(Reference)
Female	-0.57	0.13	-4.35	**<.001**	-0.57 (-0.83 ~ -0.31)	-0.32	0.18	-1.81	0.071	-0.32 (-0.67 ~ 0.03)
Age	-0.03	0.01	-6.85	**<.001**	-0.03 (-0.04 ~ -0.02)	-0.04	0.01	-4.70	**<.001**	-0.04 (-0.05 ~ -0.02)
DBP	0.02	0.01	2.99	**0.003**	0.02 (0.01 ~ 0.03)	-0.00	0.01	-0.35	0.723	-0.00 (-0.01 ~ 0.01)
SBP	0.00	0.00	0.62	0.539	0.00 (-0.00 ~ 0.01)					
BMI	0.12	0.02	7.65	**<.001**	0.12 (0.09 ~ 0.15)	0.07	0.02	3.13	**0.002**	0.07 (0.03 ~ 0.12)
WC	0.03	0.00	6.03	**<.001**	0.03 (0.02 ~ 0.03)	0.00	0.01	0.16	0.871	0.00 (-0.01 ~ 0.02)
Diabetic duration	-0.01	0.00	-2.21	**0.028**	-0.01 (-0.99 ~ -0.01)	0.00	0.00	0.49	0.625	0.00 (-0.00 ~ 0.00)
HbA1c	0.02	0.03	0.51	0.611	0.02 (-0.04 ~ 0.07)					
Hb	0.01	0.00	3.83	**<.001**	0.01 (0.01 ~ 0.02)	0.00	0.00	0.09	0.931	0.00 (-0.01 ~ 0.01)
ALT	0.01	0.00	2.19	**0.029**	0.01 (0.01 ~ 0.01)	-0.00	0.00	-0.18	0.860	-0.00 (-0.00 ~ 0.00)
AST	0.00	0.00	0.78	0.438	0.00 (-0.00 ~ 0.01)					
ALP	-0.01	0.00	-3.59	**<.001**	-0.01 (-0.01 ~ -0.01)	-0.01	0.00	-2.21	**0.028**	-0.01 (-0.99 ~ -0.01)
BUN	-0.10	0.03	-3.27	**0.001**	-0.10 (-0.17 ~ -0.04)	-0.11	0.04	-2.71	**0.007**	-0.11 (-0.19 ~ -0.03)
Cr	0.00	0.00	1.56	0.120	0.00 (-0.00 ~ 0.01)					
eGFR	0.01	0.00	2.56	**0.011**	0.01 (0.01 ~ 0.01)	-0.02	0.01	-3.64	**<.001**	-0.02 (-0.03 ~ -0.01)
UACR	0.00	0.00	0.60	0.552	0.00 (-0.00 ~ 0.00)					
UA	0.01	0.00	3.65	**<.001**	0.01 (0.01 ~ 0.01)	-0.00	0.00	-0.36	0.718	-0.00 (-0.00 ~ 0.00)
TG	0.02	0.03	0.92	0.357	0.02 (-0.03 ~ 0.07)					

Variables	Univar. Reg.					MV Reg.				
	β	S.E	t	*P*	β (95%CI)	β	S.E	t	*P*	β (95%CI)
TC	0.11	0.05	2.33	**0.020**	0.11 (0.02 ~ 0.21)	0.03	0.07	0.42	0.675	0.03 (-0.11 ~ 0.17)
HDLc	-0.57	0.24	-2.33	**0.020**	-0.57 (-1.05 ~ -0.09)	0.03	0.26	0.11	0.911	0.03 (-0.48 ~ 0.54)
LDLc	0.19	0.07	2.56	**0.011**	0.19 (0.04 ~ 0.34)	0.06	0.11	0.56	0.578	0.06 (-0.15 ~ 0.27)
HOMA-IR	-0.00	0.00	-0.48	0.632	-0.00 (-0.01 ~ 0.01)					
HOMA-β	-0.00	0.00	-1.23	0.220	-0.00 (-0.00 ~ 0.00)					
ABL	-0.17	0.57	-0.29	0.771	-0.17 (-1.28 ~ 0.95)					
baPWV	-3.63	0.76	-4.81	**<.001**	-3.63 (-5.11 ~ -2.15)	-0.88	0.93	-0.95	0.343	-0.88 (-2.71 ~ 0.94)
PTH	-0.00	0.00	-0.91	0.366	-0.00 (-0.01 ~ 0.00)					
N-t OC	-0.05	0.01	-4.28	**<.001**	-0.05 (-0.08 ~ -0.03)	-0.02	0.02	-0.96	0.338	-0.02 (-0.06 ~ 0.02)
TPINP	-0.01	0.00	-2.61	**0.009**	-0.01 (-0.01 ~ -0.01)	0.00	0.01	0.35	0.728	0.00 (-0.01 ~ 0.01)
β-CTX	-0.01	0.00	-3.98	**<.001**	-0.01 (-0.99 ~ -0.01)	-0.01	0.00	-2.55	**0.011**	-0.01 (-0.99 ~ -0.01)
25 (OH)D	-0.00	0.01	-0.45	0.651	-0.00 (-0.03 ~ 0.02)					
Ca	0.56	0.55	1.01	0.312	0.56 (-0.52 ~ 1.63)					
P	-0.00	0.00	-0.20	0.839	-0.00 (-0.01 ~ 0.01)					
